# Impact of biologic induction dose and concomitant drugs on anti‐drug antibody formation in a pediatric IBD cohort with high Hispanic representation

**DOI:** 10.1002/jpr3.70036

**Published:** 2025-05-30

**Authors:** Kenneth Grant, Jenilee Pohle, Robert Tran, Roy Nattiv

**Affiliations:** ^1^ Department of Pediatrics University of California Irvine California USA; ^2^ MemorialCare Miller Women's and Children's Hospital Long Beach California USA

**Keywords:** concomitant immunomodulators, health disparities, pediatric ulcerative colitis, social determinants of health

## Abstract

**Objectives:**

The use of biologic therapy is increasing in pediatric patients with inflammatory bowel disease (IBD). However, efficacy may be compromised by increased drug clearance and anti‐drug antibodies (ADAs). Historically, concomitant immunomodulator therapy (CIT) has been used to prevent ADA formation. Pediatric studies evaluating CIT have focused largely on white, non‐Hispanic patients and have demonstrated variable benefits. This study evaluated the utility of CIT in preventing ADAs in a pediatric IBD population with high Hispanic representation.

**Methods:**

We reviewed the charts of patients undergoing biologic induction at the Miller Children's Pediatric IBD Center between 2013 and 2023. Patients with missing therapeutic drug monitoring data or incomplete follow‐up were excluded. Categorical variables were compared using chi‐square or Fisher's exact tests, and continuous variables were analyzed using *t* tests. Multivariate logistic regression identified independent predictors of ADA formation.

**Results:**

Of 163 pediatric patients, 75 had Crohn's disease (CD) and 80 had ulcerative colitis (UC). High‐dose infliximab (IFX) was protective against ADA formation (*p* < 0.001), as were corticosteroids (*p* = 0.029). UC patients were more likely to receive corticosteroids at induction (CD: 27/75, UC: 45/80; *p* = 0.011). Among UC patients, CIT reduced ADA formation (odds ratio: 0.18; 95% confidence interval: 0.04–0.73; *p* = 0.031). Hispanic patients were more likely to have UC (CD: 18/73, UC: 46/80; *p* < 0.001). Hispanic UC patients had shorter times to biologic initiation (*p* = 0.025) and were more likely to receive both high‐dose IFX and corticosteroids at the time of biologic induction (*p* = 0.044).

**Conclusions:**

High‐dose IFX may obviate the need for CIT in ADA prevention in pediatric IBD. UC patients may benefit more from CIT than CD patients. Disparities in treatment timing and medication use among Hispanic UC patients highlight the need for further investigation.

## Abstract (highlights)

**What is Known:**

- Pediatric studies evaluating the use of concomitant immunomodulator therapy (CIT) during biologic induction for inflammatory bowel disease (IBD) have demonstrated variable benefits.
- CIT during biologic induction confers increased risk of opportunistic infection and lymphoma.
- Hispanic pediatric patients with IBD are underrepresented in large clinical studies.

**What is New:**

- High‐dose infliximab or concomitant corticosteroids at induction may obviate the need for CIT in prevention of anti‐drug antibodies.
- Pediatric patients with ulcerative colitis (UC) may benefit more from CIT than Crohn's disease patients.
- Hispanic pediatric patients with UC undergoing biologic induction may be at risk of being treated with higher levels of immunosuppression.

## 1 INTRODUCTION

Inflammatory bowel disease (IBD) is a chronic inflammatory condition characterized by two phenotypically distinct conditions, Crohn's disease (CD) and ulcerative colitis (UC). It is estimated that more than 100,000 children in the United States are affected by IBD, with the global incidence increasing annually.^1^, ^2^ Biologic agents such as infliximab (IFX) are increasingly used in pediatric IBD, with up to 55.5% of patients receiving biologics, with a mean time to initiation of 21.5 months.^3^ Despite their increased use and relative success, many patients lose response to biologics owing to the development of anti‐drug antibodies (ADAs) that increase drug clearance and decrease drug durability. Historically, concomitant immunomodulator therapy (CIT; azathioprine, 6‐mercaptopurine, and methotrexate) at the time of biologic drug induction has been employed to decrease the risk of developing ADAs.^4^ It has been suggested that CIT works by preventing the formation of ADAs, thereby improving drug durability.^5^ Studies evaluating the use of CIT in pediatric patients with IBD have demonstrated variable benefit as it relates to the development of ADAs or improved clinical outcomes.^6^, ^7^, ^8^ It has also been demonstrated that CIT with thiopurines is associated with a small but statistically significant increased risk of hepatosplenic T‐cell lymphoma, particularly in young male patients.^9^ As such, many pediatric gastroenterologists have avoided CIT when initiating biologic therapies.

Studies evaluating the use of CIT in pediatric IBD have focused on a largely homogeneous white, non‐Hispanic demographic.^6^, ^7^, ^8^ More recently, certain HLA DQ1 polymorphisms have been identified as predictors of drug immunogenicity, suggesting a genetic contribution to ADA development. While race and ethnicity encompass both genetic variability and nongenetic factors, including social determinants of health (SDOH), they have been associated with differences in IBD phenotype.^10^, ^11^ As such, they may also influence ADA formation and drug durability, warranting further investigation in understudied populations.

Our institution's physicians use CIT during biologic induction, which is also variable. Approximately half of pediatric gastroenterologists routinely utilize CIT, and the other half do not routinely utilize it. We performed a retrospective cohort study of pediatric patients undergoing biologic induction at the Miller Children's Pediatric IBD Center between 2013 and 2023 to evaluate the relationship between CIT and ADA formation. The study also examined the potential impact of patient demographics, biologic dosing, and additional concomitant medications.

## 2 METHODS

### 2.1 Ethics statement

This study was approved by the Miller Children's Hospital Institutional Review Board.

### 2.2 Patient population and variables

We queried the pediatric IBD master list at Miller Children's Hospital for patients diagnosed between October 2013 and October 2023. We included all patients who underwent induction with a biologic drug and were assessed for biologic and anti‐drug levels at least once during the study period. Patients who did not complete the induction or did not undergo therapeutic drug monitoring (TDM) on at least one occasion were excluded. Patient demographics, including age at diagnosis, sex at birth, race, and ethnicity, were recorded. Race and ethnicity were categorized according to National Institutes of Health guidelines, adhering to the Inclusion of Women, Minorities, and Children's policies. In addition, we recorded the disease phenotypes (CD, UC, and IBD‐unclassified [IBD‐U]). While patients with IBD‐U were included in the overall cohort, they were excluded from comparative analyses between CD and UC due to small sample size and heterogeneous disease phenotype. We also recorded the time from diagnosis to first biologic induction, biologic dose, and available TDM data. For the purposes of this study, high‐dose IFX was defined as induction dosing greater than 7.5 mg/kg. This threshold was selected to account for pharmacy rounding practices, where doses intended to reach ≥10 mg/kg may fall slightly below due to rounding. For patients who discontinued the first biologic therapy and underwent induction with a second biologic therapy during the study period, the time to the first biologic discontinuation and the second biologic drug induction was also recorded. The second biologic type, dose, and TDM were also recorded. Concomitant therapies, including corticosteroids, immunomodulators, and mesalamine, administered during the first and second biologic drug induction, were recorded. Patient data were manually abstracted, free of identifiers, stored on a secure computer, and tabulated using Microsoft Excel (version 16.7, Microsoft Corporation).

### 2.3 Statistical analysis

Descriptive statistics, including demographic and clinical parameters, were generated independently for all CD and UC patients (mean, median, range, standard deviation, percentage, and 95% confidence interval [CI]). Comparisons were made among groups, including age at diagnosis, sex assigned at birth, race, ethnicity, disease phenotype, biologic type, concomitant therapies, time from diagnosis to first biologic induction, time to TDM, drug level, and presence of ADAs. For survivorship analyses, patients with incomplete data (e.g., lost to follow‐up or transitioned to care outside our practice during the study period) were censored. Multivariate logistic regression analysis was performed to evaluate the risk of ADA formation using the following independent variables: age, sex, IFX versus non‐IFX induction, concomitant corticosteroids, and CIT at induction. Statistical analyses, including the chi‐square test, Fisher's exact test, *t* test, Cox‐Hazard ratio, and multiple logistic regression analyses, were performed using GraphPad Prism statistical software (version 10.1, GraphPad Software). Results were considered statistically significant at *p* < 0.05, and results with *p* ≥ 0.05 were considered not significant (NS).

## 3 RESULTS

We identified 163 pediatric patients with IBD with a median age of 13.85 at the time of diagnosis (interquartile range: 10.78–15.84) who underwent biologic induction during the study period. There were no differences in sex assigned at birth or median age at diagnosis between patients with CD and UC. Among patients who reported ethnicity, UC patients were more likely to be of Hispanic ethnicity (CD: 18/73, UC: 46/80; *p* < 0.001; Table 1).

**Table 1:** Demographics and clinical data by disease phenotype.

	Crohn's *N* = 75 (47%)	UC *N* = 80 (49%)	*p*
Age	14.0 (10.9–15.8)	13.9 (10.7–16.4)	0.581
Gender (sex assigned at birth)	Female: 24 (32) Male: 51 (68)	Female: 35 (44) Male: 45 (56)	0.140
Hispanic, Chicano(a), Latino(a)	18 (25)	46 (58)	<0.001
IFX	55 (74)	64 (80)	0.098
<7.5 mg/kg	36 (65)	32 (50)	
≥7.5 mg/kg	19 (35)	32 (50)	
Corticosteroids at induction	27 (38)	45 (56)	0.012
CIT (MTX, 6MP, AZA) at induction	29 (39)	36 (45)	0.425
Median duration of CIT (Days)	377	501	0.192
Median time to first TDM (days)	63.9 (41.6–198)	51.2 (37.3–99.4)	0.046
Median IFX level at first TDM (µg/mL)	10.3 (5.40–20.4)	10.4 (4.83–22.8)	0.962
Any anti‐drug Ab (ADA)	19 (25)	13 (16)	0.172
Median time to ADA formation (days)	548 (126–945)	148 (45.7–547)	0.071

*Note*: Data are presented as total number (%) and median (interquartile range).

Abbreviations: 6MP, 6‐mercaptopurine; ADA, anti‐drug antibody; AZA, azathioprine; CIT, concomitant immunomodulator therapy; CS, corticosteroids; IFX, infliximab; MTX, methotrexate; TDM, therapeutic drug monitoring; UC, ulcerative colitis.

Among first‐time inductors, there was no difference in the median time from diagnosis to first biologic induction between CD and UC patients (CD: 54.8 days vs. UC: 50.2 days; NS). Most patients underwent IFX induction (CD: 74% vs. UC: 80%). High‐dose IFX induction (>7.5 mg/kg) was more frequent in patients with UC than in those with CD, although the difference did not reach statistical significance (50% vs. 35%; *p* = 0.098; Table 1). In evaluating the use of concomitant therapies, patients with UC were more likely to receive corticosteroids at the time of induction (*p* = 0.012; Table 1). However, CIT utilization was similar between the two groups (CD: 39% vs. UC: 45%; NS), and the most common immunomodulator was methotrexate (CD: 55% vs. UC: 61%; NS). The median duration of immunomodulator use following induction did not differ between the CD and UC groups.

**Figure 1:**
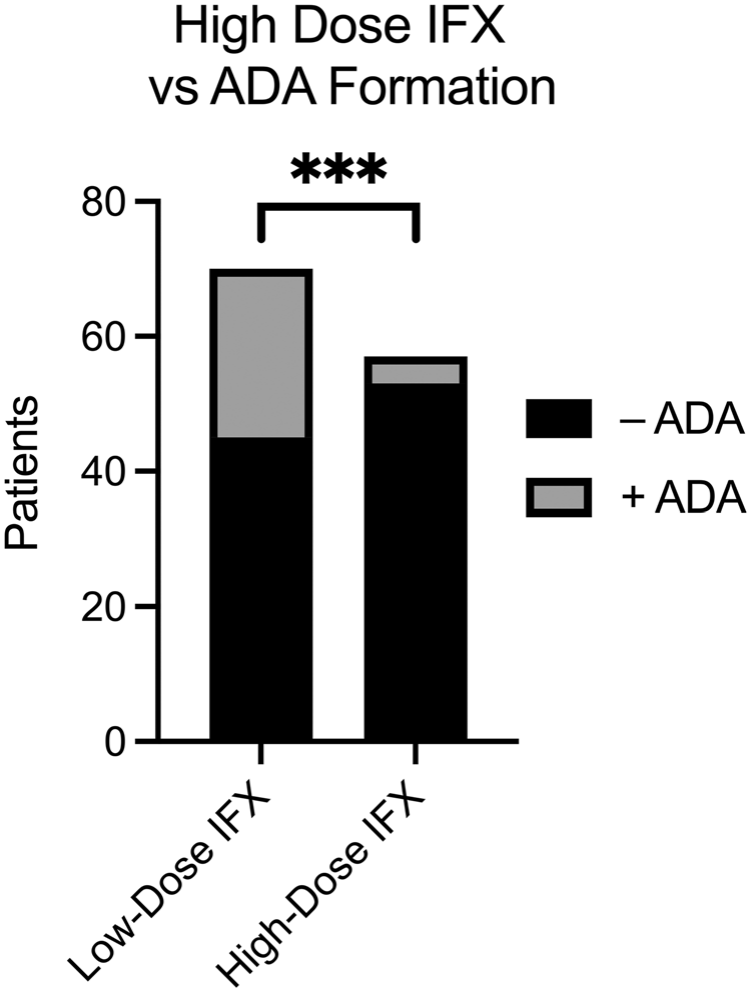
High‐dose (≥7.5 mg/kg) and low‐dose IFX (<7.5 mg/kg) induction versus ADA formation. ADA, anti‐drug antibody; IFX, infliximab.

In our cohort, age, sex, race, ethnicity, and disease phenotype were not associated with an increased risk of ADA development. The median time to the first TDM was longer in patients with CD than in those with UC (*p* = 0.046; Table 1). However, the biologic drug levels and overall rates of ADA formation were similar during the study period. Among all the patients, IFX inductors receiving high‐dose IFX appeared to be protected against ADA formation (*p* < 0.001; Figure 1). In addition, patients receiving corticosteroids at the time of biologic induction, regardless of disease phenotype or biologic prescription, were less likely to develop ADAs (*p* = 0.029; Figure S1).

As a large percentage of our population was of Hispanic ethnicity, we sought to further assess the differences between Hispanic and non‐Hispanic patients in relation to biologic drug dosing, concomitant therapies, and TDM. We observed no significant difference in time from diagnosis to first biologic induction, IFX dosing at induction or time to first TDM when comparing patients of Hispanic and non‐Hispanic ethnicity, independent of disease phenotype. Among UC patients, however, the time from diagnosis to first biologic induction was shorter for Hispanic patients compared to non‐Hispanic patients (Hispanic: 9.64 days vs. non‐Hispanic: 81.1 days; *p* = 0.025). Although the proportion of Hispanic patients with UC receiving high‐dose IFX (Hispanic: 59% vs. non‐Hispanic: 36%) and concomitant corticosteroids (Hispanic: 61% vs. non‐Hispanic: 50%) at induction appeared higher, these differences did not reach statistical significance. However, significantly more Hispanic patients received both high‐dose IFX and concomitant corticosteroids at induction compared to non‐Hispanic IFX inductors (Hispanic: 36% vs. non‐Hispanic: 12%; *p* = 0.044).

**Figure 2:**
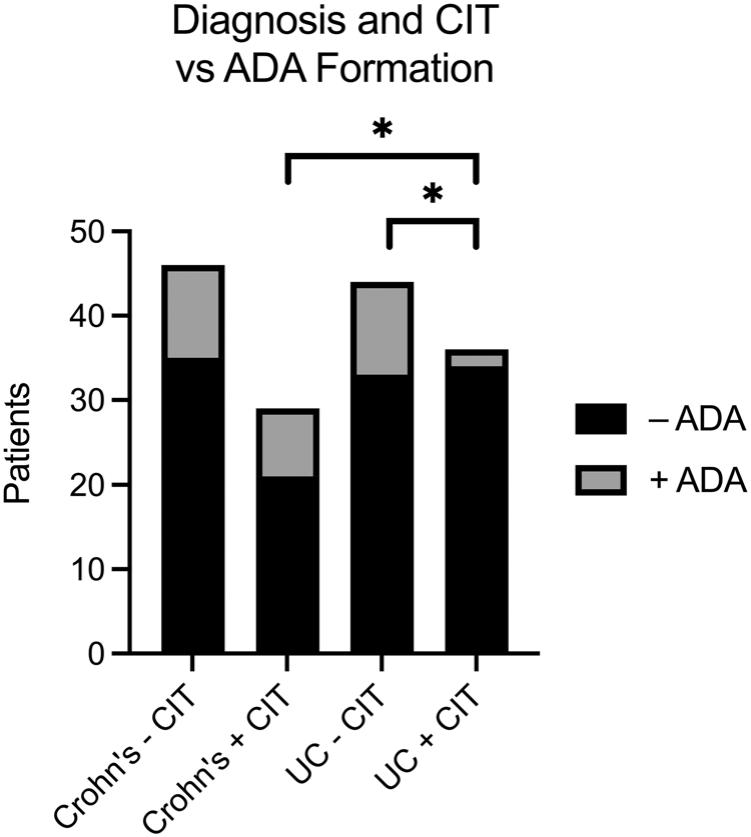
Diagnosis and CIT utilization during induction versus ADA formation. ADA, anti‐drug antibody; CIT, concomitant immunomodulator therapy.

**Figure 3:**
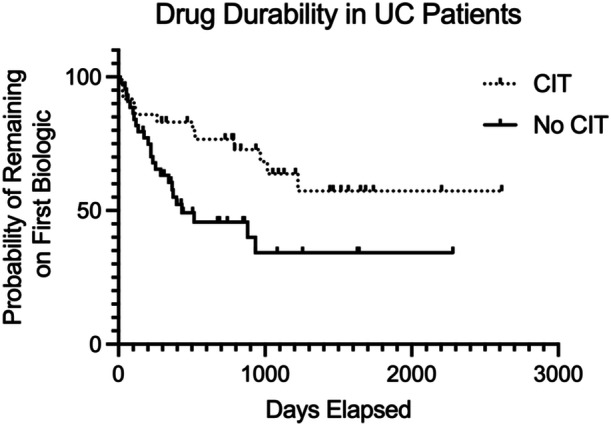
Cox‐hazard analysis demonstrating drug durability in UC patients with and without CIT. CIT, concomitant immunomodulator therapy; UC, ulcerative colitis.

Across all IBD patients, regardless of the biologic agent, CIT did not appear to be protective against ADAs. Similarly, the duration of CIT exposure did not appear to be associated with any increase or decrease in the risk of ADA formation. Compared to CD patients receiving CIT, UC patients receiving CIT were less likely to develop ADAs (odds ratio [OR]: 0.15; 95% CI: 0.03–0.78; *p* = 0.019; Figure 2). In addition, among patients with UC alone, those receiving CIT were less likely to develop ADAs (OR: 0.18; 95% CI: 0.04–0.73; *p* = 0.031; Figure 2). Multivariate logistic regression analysis of UC patients demonstrated that CIT was the only variable predictive of decreased ADA formation (*p* = 0.015). Conversely, multivariate logistic regression analysis of patients with CD demonstrated that non‐IFX biologic induction was the only variable predictive of decreased ADA formation (*p* = 0.007). Surprisingly, CIT use in patients with CD was associated with an increased risk of developing ADAs (*p* = 0.015). A Cox‐Hazard analysis demonstrated that UC inductors receiving CIT remained on their first biologic for a longer period of time than non‐CIT‐exposed patients (*p* < 0.013; Figure 3). This benefit was not observed in CD patients receiving CIT (Figure S2).

## 4 DISCUSSION

This study demonstrates that high‐dose IFX and concomitant corticosteroid use at the time of biologic induction in pediatric patients with IBD is associated with decreased formation of ADAs. In addition, UC patients appear to benefit from CIT, as evidenced by lower ADA formation compared to CD patients receiving CIT.

At our institution, the use of high‐dose IFX, defined as induction dosing of >7.5 mg/kg, was associated with decreased ADA formation. This finding aligns with previously published data demonstrating that accelerated induction dosing of IFX guided by pharmacokinetic dashboards, to achieve more robust levels, improves drug durability and reduces immunogenicity.^12^ Although high‐dose IFX has been routinely used by pediatric gastroenterologists for over a decade, insurance carriers frequently cite a lack of pediatric‐specific data to support its use in pediatric IBD.^13^ This study adds to the body of literature supporting the use of high‐dose IFX during induction therapy in pediatric patients with IBD.

The use of CIT at the time of biologic induction in the treatment of IBD has been shown to decrease the risk of ADA formation and improve therapeutic drug levels and drug durability in both adult and pediatric populations.^4^, ^7^, ^8^ For instance, Hoelz et al. demonstrated that early CIT within 3 months of biologic induction did not reduce ADA formation but significantly decreased loss of response (LOR) compared to delayed CIT initiation or biologic monotherapy. While this study included analyses based on disease phenotype, its primary focus was on the general IBD population rather than exploring disease‐specific differences in ATI formation and LOR. Kappelman et al. evaluated pediatric patients with CD undergoing induction with either IFX or adalimumab and concluded that CIT did not significantly decrease ADA formation overall. However, adalimumab‐treated patients receiving CIT exhibited a significant reduction in LOR. Despite these findings, CIT is often used cautiously due to concerns about immunosuppression and lymphoma risk with thiopurines. Consequently, some pediatric gastroenterologists prefer monotherapy. The Trough Concentration Adapted Infliximab Treatment trial demonstrated that proactive drug monitoring and dose adjustments could reduce ADAs and obviate the need for CIT.^14^

At our institution, approximately half of pediatric gastroenterologists routinely utilize CIT during biologic induction, while the other half prefer monotherapy. This variability provided a unique opportunity to evaluate the real‐world impact of CIT on ADA formation and drug durability in pediatric IBD patients.

Similar to previously published findings, our data indicate that CIT did not confer a protective effect against ADAs when evaluating the entire IBD cohort. However, when stratified by disease phenotype, our data suggest that UC patients, in particular, benefit from CIT, with a significantly lower likelihood of developing ADAs compared to their non‐CIT counterparts. Conversely, CD patients receiving CIT not only failed to show protection against ADAs but also exhibited a paradoxical increase in ADA risk. These findings highlight potential differences in immunogenicity between CD and UC populations, raising questions about disease‐specific mechanisms influencing ADA formation.

It is notable that our UC population showed a trend toward higher dose at induction and decreased time to first TDM compared to the CD population, suggesting that the UC population was perhaps treated more aggressively around the time of induction and was able to achieve therapeutic levels more quickly. However, among patients with UC alone, those receiving CIT also appeared to be more protected against ADAs than patients with UC who did not receive CIT. Multivariate logistic regression analysis of patients with UC demonstrated that CIT was the only beneficial parameter in preventing ADAs in our cohort. UC patients receiving CIT also experienced improved drug durability compared with their non‐CIT counterparts. In contrast, multivariate logistic regression modeling of our CD cohort demonstrated that non‐IFX induction was associated with decreased ADA formation. Again, this may be explained by a relatively higher proportion of low‐dose IFX induction and a longer time to first TDM in our CD population, which may have led to an increased risk for the development of ADAs. Another interesting finding in the multivariate analysis in our CD cohort was that CIT was paradoxically associated with an increased risk of ADA. We hypothesize that poor compliance with CIT medications in select patients may have contributed to increased ADA risk and confounded the multivariate analysis of our CD population. We did not assess compliance or thiopurine levels, which is a limitation of the present study.

Studies examining the use of CIT during biologic induction in pediatric IBD have focused on general efficacy and safety rather than directly comparing the outcomes between UC and CD patients.^7^ Although the benefits of CIT have been generally considered applicable to both UC and CD patients, our study suggests that only UC patients benefited from decreased immunogenicity.

Recently, several groups have uncovered genotypic differences that may put patients at risk of developing ADAs.^15^ This suggests a potential hereditary predisposition, where specific genetic variants potentially passed along or concentrated within specific ethnic groups, could influence the immunogenic response to the therapy. We did not review the HLADQA1*05 polymorphic risk alleles, which is also a limitation of this study. However, race and ethnicity did not appear to be directly associated with an increased risk of developing ADAs in our study.

Our IBD program is home to a relatively large number of Hispanic patients, which is a largely understudied and underrepresented demographic in large clinical trials. Recent literature suggests that Hispanic patients with IBD have a relatively younger age of onset, making them a particularly vulnerable population.^16^ Studies evaluating IBD phenotype in Hispanic populations have yielded conflicting results.^17^, ^18^ In our cohort, Hispanic patients were more likely to present with UC, and among UC patients, they had a significantly shorter time to first biologic induction compared to non‐Hispanic patients. While a higher proportion of Hispanic patients received either high‐dose IFX or concomitant corticosteroids during induction, these differences were not statistically significant. However, Hispanic patients with UC were significantly more likely to receive both high‐dose IFX and concomitant corticosteroids at the time of induction compared to non‐Hispanic patients. While these findings may reflect true phenotypic differences, our study was limited by a lack of detailed clinical, endoscopic, and histologic data, as well as the absence of genetic and pharmacokinetic analyses. Additionally, we did not assess the impact of SDOH or potential systemic biases in treatment approaches, both of which may contribute to differences in medication use and timing of therapy. Future research should integrate these factors to better understand disparities in disease course and treatment response among Hispanic pediatric IBD patients.

## 5 CONCLUSION

Our findings support the use of high‐dose IFX at biologic induction to reduce ADA formation and suggest that UC patients may derive additional benefits from CIT. However, CD patients may not experience the same degree of protection from CIT, indicating a potential difference in disease biology or treatment response. Multi‐center collaborations are needed to validate these findings, incorporate genetic profiling to explore ADA risk factors, and develop strategies to optimize induction regimens for diverse patient populations. Future studies should also prioritize understanding the impact of SDOH on sustained remission, hospitalizations, surgeries, and quality of life in underrepresented populations.

## CONFLICT OF INTEREST STATEMENT

The authors declare no conflicts of interest.

## Supporting information

Supplemental Figure_1. Concomitant CS at time of biologic induction versus ADA formation. CS: Corticosteroids. ADA: Anti‐drug antibodies.

Supplemental Figure_2. Cox‐Hazard Analysis demonstrating drug durability in CD patients with and without CIT. CD: Crohn's disease. CIT: Concomitant immunomodulator therapy.
