# Supplementary figures and images for: Impact of biologic induction dose and concomitant drugs on anti‐drug antibody formation in a pediatric IBD cohort with high Hispanic representation
Source: JPGN Rep. 2025 May 30;6(4):437–43. doi: 10.1002/jpr3.70036 (PMC12611574; doi:10.1002/jpr3.70036)

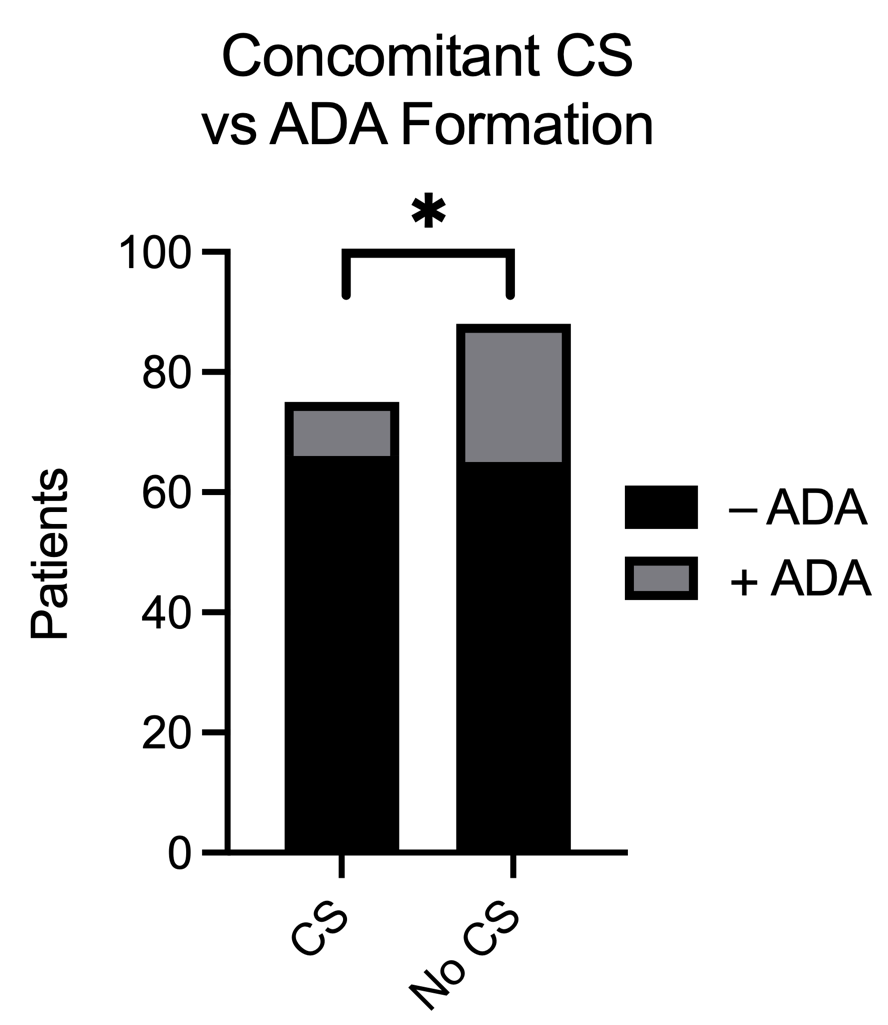

Supplement: Supplementary file 1 — Supplemental Figure_1. Concomitant CS at time of biologic induction versus ADA formation. CS: Corticosteroids. ADA: Anti‐drug antibodies. [file JPR3-6-437-s001.tiff]

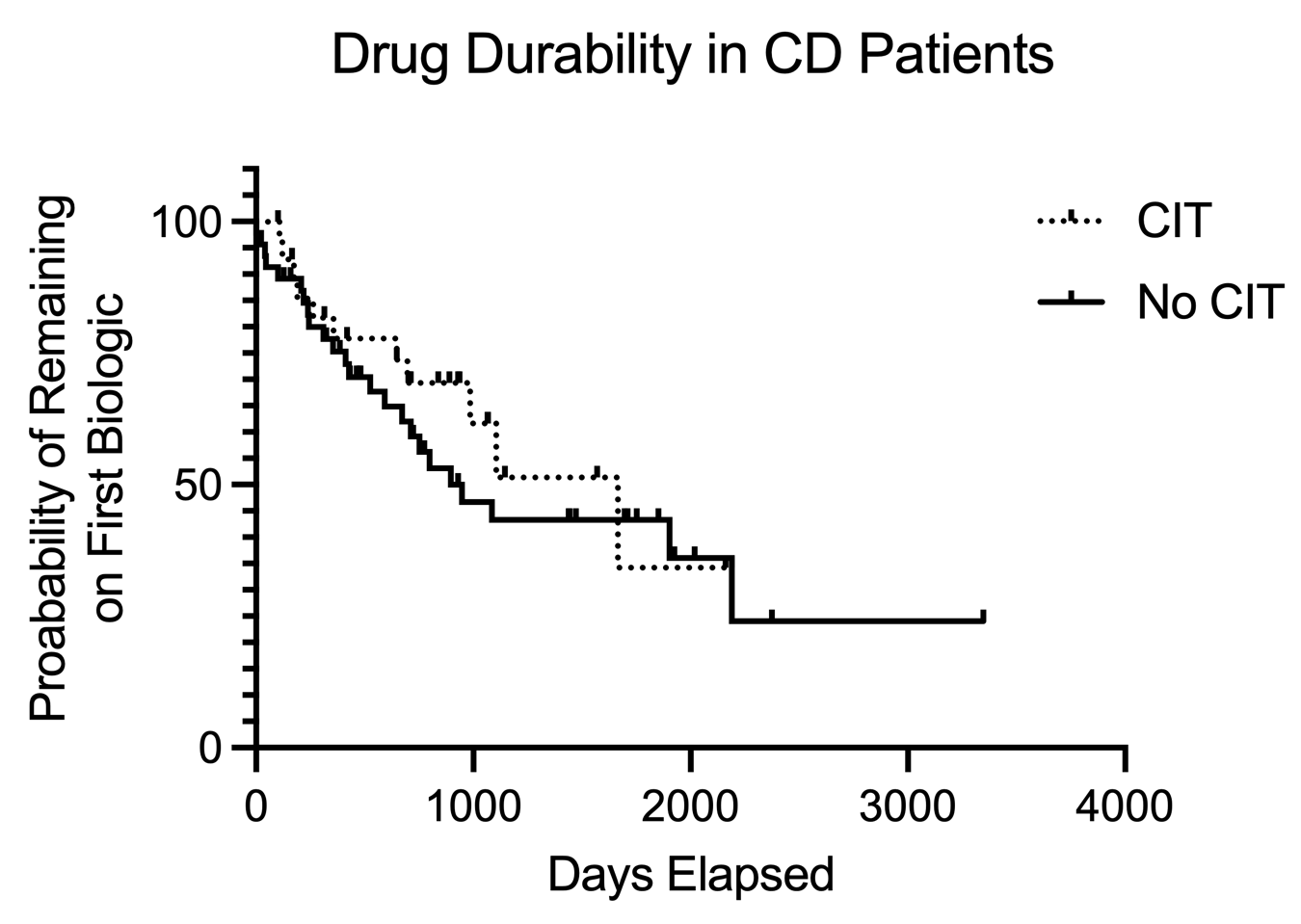

Supplement: Supplementary file 2 — Supplemental Figure_2. Cox‐Hazard Analysis demonstrating drug durability in CD patients with and without CIT. CD: Crohn's disease. CIT: Concomitant immunomodulator therapy. [file JPR3-6-437-s002.tiff]
